# 3-Acetyl-1-phenyl­thio­urea

**DOI:** 10.1107/S1600536812002371

**Published:** 2012-01-25

**Authors:** Durre Shahwar, M. Nawaz Tahir, Muhammad Mansha Chohan, Naeem Ahmad, Samiullah

**Affiliations:** a Department of Chemistry, Government College University, Lahore, Pakistan; b University of Sargodha, Department of Physics, Sargodha, Pakistan

## Abstract

In the crystal structure of title compound, C_9_H_10_N_2_OS, there are two symmetry-independent mol­ecules, each having an intra­molecular N—H⋯O hydrogen bond generating an *S*(6) ring motif. The benzene rings and the virtually planar acetyl­thoiurea fragments [r.m.s. deviations = 0.0045 and 0.0341 Å] are oriented at dihedral angles of 50.71 (6) and 62.79 (6)° in the two mol­ecules. In the crystal, N—H⋯S and N—H⋯O hydrogen bonds link mol­ecules *via* cyclic *R*_2_^2^(8) and *R*_2_^2^(12) motifs into a one-dimensional polymeric network extending along [101]. The intra- and inter­molecular N—H⋯O inter­actions are part of a three-center hydrogen bond. A C—H⋯S inter­action also occurs.

## Related literature

For related structures, see: Othman *et al.* (2010 ▶). For graph-set notation, see: Bernstein *et al.* (1995 ▶).
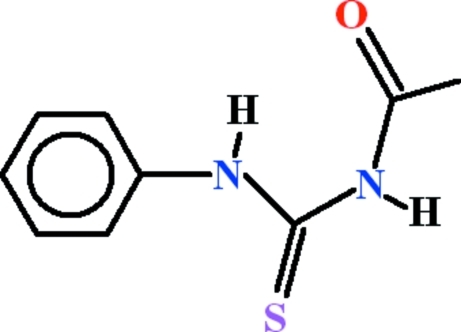


## Experimental

### 

#### Crystal data

C_9_H_10_N_2_OS
*M* *_r_* = 194.25
Monoclinic, 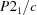

*a* = 10.1911 (2) Å
*b* = 22.5480 (4) Å
*c* = 8.9736 (2) Å
β = 112.449 (1)°
*V* = 1905.77 (7) Å^3^
*Z* = 8
Mo *K*α radiation
μ = 0.30 mm^−1^
*T* = 296 K
0.35 × 0.25 × 0.22 mm

#### Data collection

Bruker Kappa APEXII CCD diffractometer
Absorption correction: multi-scan (*SADABS*; Bruker, 2005 ▶) *T*_min_ = 0.915, *T*_max_ = 0.938
16643 measured reflections
4679 independent reflections
3626 reflections with *I* > 2σ(*I*)
*R*_int_ = 0.024

#### Refinement

*R*[*F* ^2^ > 2σ(*F* ^2^)] = 0.045
*wR*(*F* ^2^) = 0.129
*S* = 1.05
4679 reflections
228 parameters
H-atom parameters constrained
Δρ_max_ = 0.30 e Å^−3^
Δρ_min_ = −0.35 e Å^−3^

### 

Data collection: *APEX2* (Bruker, 2009 ▶); cell refinement: *SAINT* (Bruker, 2009 ▶); data reduction: *SAINT*; program(s) used to solve structure: *SHELXS97* (Sheldrick, 2008 ▶); program(s) used to refine structure: *SHELXL97* (Sheldrick, 2008 ▶); molecular graphics: *ORTEP-3 for Windows* (Farrugia, 1997 ▶) and *PLATON* (Spek, 2009 ▶); software used to prepare material for publication: *WinGX* (Farrugia, 1999 ▶) and *PLATON*.

## Supplementary Material

Crystal structure: contains datablock(s) global, I. DOI: 10.1107/S1600536812002371/gk2451sup1.cif

Structure factors: contains datablock(s) I. DOI: 10.1107/S1600536812002371/gk2451Isup2.hkl

Supplementary material file. DOI: 10.1107/S1600536812002371/gk2451Isup3.cml

Additional supplementary materials:  crystallographic information; 3D view; checkCIF report

## Figures and Tables

**Table 1:** Hydrogen-bond geometry (Å, °)

*D*—H⋯*A*	*D*—H	H⋯*A*	*D*⋯*A*	*D*—H⋯*A*
N1—H1⋯O1	0.86	1.97	2.662 (2)	136
N1—H1⋯O2^i^	0.86	2.46	3.1967 (19)	143
N2—H2*A*⋯S2^ii^	0.86	2.64	3.4931 (17)	170
N3—H3*A*⋯O2	0.86	1.97	2.6633 (7)	137
N3—H3*A*⋯O1^i^	0.86	2.42	3.1418 (18)	142
N4—H4*A*⋯S1^ii^	0.86	2.57	3.4150 (18)	168
C18—H18*B*⋯S1^ii^	0.96	2.83	3.594 (3)	137

Symmetry codes: (i) 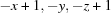; (ii) 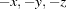.

**Fig. 1.**
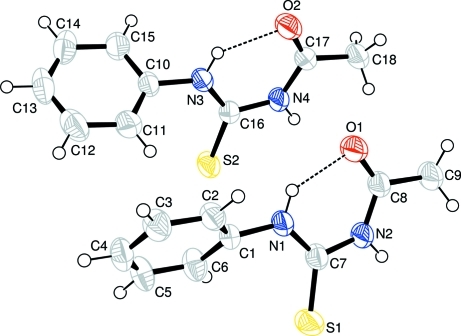
View of the title compound with the displacement ellipsoids drawn at the 50% probability level. H atoms are shown by small circles of arbitrary radii. The dotted lines represent the intramolecular H-bondings.

**Fig. 2.**
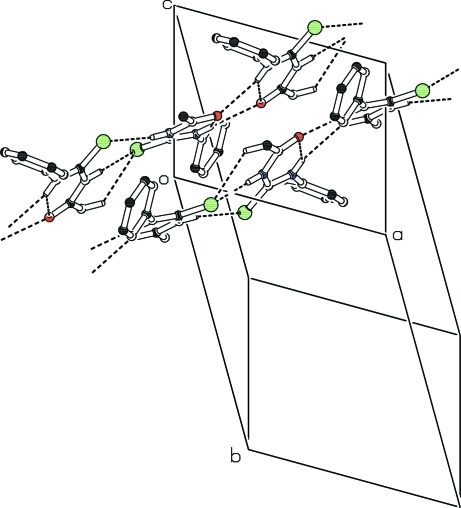
The partial packing (PLATON; Spek, 2009) which shows that molecules form polymeric chains extending along [1 0 1] direction.

## supplementary crystallographic information

### Comment

The title compound I (Fig. 1) has been synthesized in search of new enzyme inhibitors.

The crystal structure of *N*-(3-chloropropionyl)-*N*'-phenylthiourea (Othman *et al.*, 2010) has been published which is related to the title compound (I).

In (I), two molecules in the asymmetric unit are present, which differ from each other geometrically. In one molecule, the benzene ring A (C1–C6) and the acetylthoiurea moiety B (N1/C7/S1/N2/C8/O1/C9) are planar with r. m. s. deviation of 0.0045 Å and 0.0341 Å, respectively. The dihedral angle between A/B is 50.71 (6)°. In second molecule, the benzene ring C (C10–C15) and the acetylthoiurea moiety D (N3/C16/S2/N4/C17/O2/C18) are also planar with r. m. s. deviation of 0.0037 Å and 0.0453 Å, respectively and the dihedral angle between C/D is 62.79 (6)°. In both molecules, there exist classical intramolecular H–bonding of N—H···O type (Table 1, Fig. 1) with *S*(6) ring motif (Bernstein *et al.*, 1995). Both molecules are interliked due to strong N—H···O, N—H···S and C—H···S types of H–bondings (Table 1, Fig. 2). The S(6) ring motifs of both molecules are connected into a four membered ring (—O···H···O···H···O—). The N—H···S type of bonding completes *R*_2_^2^(8) ring motif. *R*_2_^1^(6) ring motif is formed due to C—H···S and N—H···S types of intermolecular H-bondings (Fig. 2). The molecules are linked to form of one-dimensional polymeric chains extending along [101] (Fig. 2).

### Experimental

The title compound was synthesized by adding 0.1 mol (7.13 ml) of acetyl chloride dropwise to a stirred solution of KSCN (0.11 mol) in dry acetone (50 ml), followed by slow addition of aniline (0.1 mol) in dry acetone (25 ml). The mixture was refluxed for 5–10 min, then poured on ice cooled water, which resulted in crude precipitate. Recrystallization of the precipitate in from ethyl acetate yielded light green prisms (m.p. 365 K).

### Refinement

The H atoms were positioned geometrically (C—H = 0.93–0.96 Å, N—H = 0.86 Å) and refined as riding with *U*_iso_(H) = *xU*_eq_(C, N), where *x* = 1.5 for methyl groups and *x* = 1.2 for other H atoms.

### Crystal data

**Table:**

C_9_H_10_N_2_OS	*F*(000) = 816
*M**_r_* = 194.25	*D*_x_ = 1.354 Mg m^−^^3^
Monoclinic, *P*2_1_/*c*	Mo *K*α radiation, λ = 0.71073 Å
Hall symbol: -P 2ybc	Cell parameters from 3626 reflections
*a* = 10.1911 (2) Å	θ = 2.2–28.3°
*b* = 22.5480 (4) Å	µ = 0.30 mm^−^^1^
*c* = 8.9736 (2) Å	*T* = 296 K
β = 112.449 (1)°	Prism, light green
*V* = 1905.77 (7) Å^3^	0.35 × 0.25 × 0.22 mm
*Z* = 8	

### Data collection

**Table:**

Bruker Kappa APEXII CCD diffractometer	4679 independent reflections
Radiation source: fine-focus sealed tube	3626 reflections with *I* > 2σ(*I*)
graphite	*R*_int_ = 0.024
Detector resolution: 8.00 pixels mm^-1^	θ_max_ = 28.3°, θ_min_ = 2.2°
ω scans	*h* = −13→13
Absorption correction: multi-scan (*SADABS*; Bruker, 2005)	*k* = −23→29
*T*_min_ = 0.915, *T*_max_ = 0.938	*l* = −11→11
16643 measured reflections	

### Refinement

**Table:**

Refinement on *F*^2^	Primary atom site location: structure-invariant direct methods
Least-squares matrix: full	Secondary atom site location: difference Fourier map
*R*[*F*^2^ > 2σ(*F*^2^)] = 0.045	Hydrogen site location: inferred from neighbouring sites
*wR*(*F*^2^) = 0.129	H-atom parameters constrained
*S* = 1.05	*w* = 1/[σ^2^(*F*_o_^2^) + (0.0574*P*)^2^ + 0.6731*P*] where *P* = (*F*_o_^2^ + 2*F*_c_^2^)/3
4679 reflections	(Δ/σ)_max_ < 0.001
228 parameters	Δρ_max_ = 0.30 e Å^−^^3^
0 restraints	Δρ_min_ = −0.35 e Å^−^^3^

### Special details

**Table:**

Geometry. Bond distances, angles *etc*. have been calculated using the rounded fractional coordinates. All su's are estimated from the variances of the (full) variance-covariance matrix. The cell e.s.d.'s are taken into account in the estimation of distances, angles and torsion angles
Refinement. Refinement of *F*^2^ against ALL reflections. The weighted *R*-factor *wR* and goodness of fit *S* are based on *F*^2^, conventional *R*-factors *R* are based on *F*, with *F* set to zero for negative *F*^2^. The threshold expression of *F*^2^ > σ(*F*^2^) is used only for calculating *R*-factors(gt) *etc*. and is not relevant to the choice of reflections for refinement. *R*-factors based on *F*^2^ are statistically about twice as large as those based on *F*, and *R*- factors based on ALL data will be even larger.

### Fractional atomic coordinates and isotropic or equivalent isotropic displacement parameters (Å^2^)

**Table:**

	*x*	*y*	*z*	*U*_iso_*/*U*_eq_	
S1	−0.19196 (6)	0.05015 (3)	0.17599 (8)	0.0666 (2)	
O1	0.22186 (16)	−0.05280 (6)	0.3471 (2)	0.0647 (5)	
N1	0.08613 (17)	0.04741 (6)	0.35398 (19)	0.0455 (5)	
N2	−0.01041 (17)	−0.03499 (6)	0.19702 (19)	0.0463 (5)	
C1	0.09330 (18)	0.10632 (7)	0.4158 (2)	0.0388 (5)	
C2	0.1603 (2)	0.11483 (8)	0.5792 (2)	0.0455 (6)	
C3	0.1800 (2)	0.17188 (9)	0.6419 (3)	0.0548 (7)	
C4	0.1317 (2)	0.21994 (8)	0.5415 (3)	0.0546 (7)	
C5	0.0635 (3)	0.21096 (8)	0.3790 (3)	0.0575 (7)	
C6	0.0449 (2)	0.15463 (8)	0.3146 (2)	0.0532 (7)	
C7	−0.0283 (2)	0.02154 (8)	0.2479 (2)	0.0428 (5)	
C8	0.1098 (2)	−0.06975 (8)	0.2494 (2)	0.0449 (6)	
C9	0.08992 (12)	−0.13043 (2)	0.17563 (12)	0.0596 (7)	
S2	0.29361 (6)	0.11538 (2)	0.07535 (6)	0.0571 (2)	
O2	0.59666 (10)	−0.02218 (2)	0.39879 (7)	0.0624 (5)	
N3	0.52470 (6)	0.09140 (2)	0.33705 (7)	0.0456 (5)	
N4	0.41578 (16)	0.01207 (6)	0.1764 (2)	0.0463 (5)	
C10	0.5529 (2)	0.15166 (7)	0.3895 (2)	0.0419 (5)	
C11	0.4580 (2)	0.18411 (10)	0.4315 (3)	0.0569 (7)	
C12	0.4945 (3)	0.24100 (10)	0.4927 (3)	0.0692 (9)	
C13	0.6232 (3)	0.26489 (9)	0.5078 (3)	0.0669 (8)	
C14	0.7165 (3)	0.23256 (10)	0.4653 (3)	0.0641 (8)	
C15	0.6826 (2)	0.17540 (9)	0.4056 (3)	0.0523 (6)	
C16	0.41954 (19)	0.07247 (7)	0.2070 (2)	0.0405 (5)	
C17	0.5029 (2)	−0.03202 (8)	0.2695 (2)	0.0459 (6)	
C18	0.4715 (2)	−0.09258 (9)	0.1962 (3)	0.0590 (7)	
H1	0.16310	0.02690	0.38876	0.0545*	
H2	0.19244	0.08241	0.64760	0.0546*	
H2A	−0.08394	−0.05025	0.12321	0.0555*	
H3	0.22598	0.17763	0.75237	0.0658*	
H4	0.14526	0.25818	0.58346	0.0655*	
H5	0.02914	0.24337	0.31114	0.0690*	
H6	0.00015	0.14915	0.20379	0.0639*	
H9A	0.15836	−0.15710	0.24765	0.0894*	
H9B	0.10276	−0.12869	0.07516	0.0894*	
H9C	−0.00410	−0.14434	0.15685	0.0894*	
H3A	0.58190	0.06497	0.39608	0.0548*	
H4A	0.35054	0.00054	0.08761	0.0556*	
H11	0.37031	0.16814	0.41908	0.0682*	
H12	0.43186	0.26299	0.52351	0.0830*	
H13	0.64664	0.30324	0.54715	0.0803*	
H14	0.80349	0.24893	0.47639	0.0769*	
H15	0.74639	0.15337	0.37671	0.0627*	
H18A	0.52816	−0.09968	0.13370	0.0886*	
H18B	0.37273	−0.09519	0.12774	0.0886*	
H18C	0.49338	−0.12174	0.28022	0.0886*	

### Atomic displacement parameters (Å^2^)

**Table:**

	*U*^11^	*U*^22^	*U*^33^	*U*^12^	*U*^13^	*U*^23^
S1	0.0461 (3)	0.0524 (3)	0.0799 (4)	0.0084 (2)	0.0003 (3)	−0.0220 (3)
O1	0.0495 (8)	0.0449 (8)	0.0774 (10)	0.0053 (6)	−0.0008 (8)	−0.0177 (7)
N1	0.0430 (8)	0.0309 (8)	0.0525 (9)	0.0012 (6)	0.0071 (7)	−0.0056 (6)
N2	0.0436 (8)	0.0339 (8)	0.0500 (9)	−0.0021 (6)	0.0053 (7)	−0.0088 (6)
C1	0.0400 (9)	0.0290 (8)	0.0459 (9)	−0.0014 (7)	0.0147 (8)	−0.0031 (7)
C2	0.0501 (10)	0.0370 (9)	0.0446 (10)	0.0048 (8)	0.0128 (8)	0.0013 (7)
C3	0.0629 (12)	0.0491 (11)	0.0465 (11)	0.0021 (9)	0.0143 (9)	−0.0117 (9)
C4	0.0709 (13)	0.0340 (10)	0.0654 (13)	−0.0038 (9)	0.0332 (11)	−0.0104 (9)
C5	0.0835 (15)	0.0314 (10)	0.0602 (12)	0.0039 (9)	0.0302 (12)	0.0064 (8)
C6	0.0739 (14)	0.0386 (10)	0.0422 (10)	0.0001 (9)	0.0166 (10)	0.0025 (8)
C7	0.0470 (10)	0.0336 (9)	0.0431 (9)	−0.0001 (7)	0.0121 (8)	−0.0024 (7)
C8	0.0472 (10)	0.0342 (9)	0.0475 (10)	0.0001 (7)	0.0115 (8)	−0.0036 (7)
C9	0.0599 (12)	0.0392 (10)	0.0691 (14)	0.0009 (9)	0.0128 (11)	−0.0143 (10)
S2	0.0581 (3)	0.0369 (3)	0.0535 (3)	0.0050 (2)	−0.0041 (2)	0.0012 (2)
O2	0.0595 (9)	0.0436 (8)	0.0599 (9)	0.0107 (7)	−0.0041 (7)	−0.0056 (7)
N3	0.0441 (8)	0.0323 (7)	0.0481 (9)	0.0042 (6)	0.0037 (7)	−0.0030 (6)
N4	0.0443 (8)	0.0328 (8)	0.0485 (9)	−0.0012 (6)	0.0029 (7)	−0.0049 (6)
C10	0.0478 (10)	0.0309 (8)	0.0381 (9)	0.0018 (7)	0.0063 (8)	−0.0013 (7)
C11	0.0540 (12)	0.0503 (12)	0.0620 (13)	0.0037 (9)	0.0174 (10)	−0.0091 (9)
C12	0.0836 (17)	0.0504 (13)	0.0657 (14)	0.0180 (12)	0.0197 (13)	−0.0105 (10)
C13	0.0972 (19)	0.0329 (10)	0.0563 (13)	−0.0030 (11)	0.0134 (12)	−0.0048 (9)
C14	0.0773 (15)	0.0446 (12)	0.0647 (14)	−0.0174 (11)	0.0206 (12)	−0.0023 (10)
C15	0.0573 (12)	0.0424 (10)	0.0543 (11)	−0.0020 (9)	0.0182 (10)	−0.0015 (8)
C16	0.0394 (9)	0.0337 (9)	0.0443 (9)	−0.0012 (7)	0.0114 (8)	−0.0004 (7)
C17	0.0412 (9)	0.0357 (9)	0.0532 (11)	0.0031 (7)	0.0095 (8)	−0.0017 (8)
C18	0.0563 (12)	0.0348 (10)	0.0703 (14)	0.0060 (9)	0.0067 (10)	−0.0063 (9)

### Geometric parameters (Å, °)

**Table:**

S1—C7	1.671 (2)	C2—H2	0.9300
S2—C16	1.6796 (18)	C3—H3	0.9300
O1—C8	1.206 (3)	C4—H4	0.9300
O2—C17	1.2090 (19)	C5—H5	0.9300
N1—C7	1.326 (2)	C6—H6	0.9300
N1—C1	1.431 (2)	C9—H9A	0.9600
N2—C7	1.389 (2)	C9—H9B	0.9600
N2—C8	1.377 (3)	C9—H9C	0.9600
N1—H1	0.8600	C10—C15	1.382 (3)
N2—H2A	0.8600	C10—C11	1.375 (3)
N3—C10	1.4307 (17)	C11—C12	1.389 (3)
N3—C16	1.3181 (18)	C12—C13	1.376 (4)
N4—C16	1.387 (2)	C13—C14	1.363 (4)
N4—C17	1.381 (2)	C14—C15	1.388 (3)
N3—H3A	0.8600	C17—C18	1.496 (3)
N4—H4A	0.8600	C11—H11	0.9300
C1—C6	1.383 (2)	C12—H12	0.9300
C1—C2	1.374 (2)	C13—H13	0.9300
C2—C3	1.388 (3)	C14—H14	0.9300
C3—C4	1.375 (3)	C15—H15	0.9300
C4—C5	1.370 (4)	C18—H18A	0.9600
C5—C6	1.378 (3)	C18—H18B	0.9600
C8—C9	1.4996 (19)	C18—H18C	0.9600
			
C1—N1—C7	125.98 (17)	H9A—C9—H9C	109.00
C7—N2—C8	128.41 (16)	C8—C9—H9A	109.00
C7—N1—H1	117.00	H9B—C9—H9C	109.00
C1—N1—H1	117.00	C8—C9—H9C	109.00
C8—N2—H2A	116.00	H9A—C9—H9B	109.00
C7—N2—H2A	116.00	C8—C9—H9B	109.00
C10—N3—C16	126.30 (11)	C11—C10—C15	120.67 (17)
C16—N4—C17	128.62 (16)	N3—C10—C11	121.43 (18)
C16—N3—H3A	117.00	N3—C10—C15	117.80 (17)
C10—N3—H3A	117.00	C10—C11—C12	119.3 (2)
C17—N4—H4A	116.00	C11—C12—C13	120.1 (2)
C16—N4—H4A	116.00	C12—C13—C14	120.2 (2)
C2—C1—C6	119.87 (15)	C13—C14—C15	120.5 (3)
N1—C1—C2	118.32 (15)	C10—C15—C14	119.2 (2)
N1—C1—C6	121.61 (15)	S2—C16—N3	125.50 (11)
C1—C2—C3	119.88 (17)	S2—C16—N4	118.07 (13)
C2—C3—C4	120.3 (2)	N3—C16—N4	116.42 (14)
C3—C4—C5	119.36 (19)	O2—C17—C18	123.18 (17)
C4—C5—C6	121.01 (18)	N4—C17—C18	114.25 (16)
C1—C6—C5	119.54 (17)	O2—C17—N4	122.57 (16)
N1—C7—N2	116.73 (18)	C10—C11—H11	120.00
S1—C7—N2	117.66 (14)	C12—C11—H11	120.00
S1—C7—N1	125.58 (14)	C11—C12—H12	120.00
O1—C8—N2	122.67 (17)	C13—C12—H12	120.00
N2—C8—C9	114.58 (15)	C12—C13—H13	120.00
O1—C8—C9	122.74 (17)	C14—C13—H13	120.00
C3—C2—H2	120.00	C13—C14—H14	120.00
C1—C2—H2	120.00	C15—C14—H14	120.00
C4—C3—H3	120.00	C10—C15—H15	120.00
C2—C3—H3	120.00	C14—C15—H15	120.00
C3—C4—H4	120.00	C17—C18—H18A	109.00
C5—C4—H4	120.00	C17—C18—H18B	109.00
C6—C5—H5	120.00	C17—C18—H18C	109.00
C4—C5—H5	119.00	H18A—C18—H18B	109.00
C1—C6—H6	120.00	H18A—C18—H18C	109.00
C5—C6—H6	120.00	H18B—C18—H18C	109.00
			
C7—N1—C1—C2	133.6 (2)	C6—C1—C2—C3	−0.4 (3)
C7—N1—C1—C6	−51.6 (3)	N1—C1—C6—C5	−175.2 (2)
C1—N1—C7—S1	−5.7 (3)	C2—C1—C6—C5	−0.5 (3)
C1—N1—C7—N2	176.57 (16)	N1—C1—C2—C3	174.48 (19)
C8—N2—C7—S1	−173.67 (16)	C1—C2—C3—C4	0.6 (3)
C8—N2—C7—N1	4.2 (3)	C2—C3—C4—C5	0.2 (4)
C7—N2—C8—O1	−3.3 (3)	C3—C4—C5—C6	−1.2 (4)
C7—N2—C8—C9	176.44 (16)	C4—C5—C6—C1	1.3 (4)
C10—N3—C16—S2	−1.0 (2)	N3—C10—C11—C12	175.39 (18)
C16—N3—C10—C11	62.9 (2)	C15—C10—C11—C12	−0.9 (3)
C16—N3—C10—C15	−120.69 (19)	N3—C10—C15—C14	−176.23 (18)
C10—N3—C16—N4	177.67 (15)	C11—C10—C15—C14	0.2 (3)
C16—N4—C17—C18	−177.93 (19)	C10—C11—C12—C13	1.3 (4)
C17—N4—C16—S2	−176.84 (17)	C11—C12—C13—C14	−1.1 (4)
C17—N4—C16—N3	4.4 (3)	C12—C13—C14—C15	0.4 (4)
C16—N4—C17—O2	1.8 (3)	C13—C14—C15—C10	0.1 (4)

### Hydrogen-bond geometry (Å, °)

**Table:**

*D*—H···*A*	*D*—H	H···*A*	*D*···*A*	*D*—H···*A*
N1—H1···O1	0.86	1.97	2.662 (2)	136
N1—H1···O2^i^	0.86	2.46	3.1967 (19)	143
N2—H2A···S2^ii^	0.86	2.64	3.4931 (17)	170
N3—H3A···O2	0.86	1.97	2.6633 (7)	137
N3—H3A···O1^i^	0.86	2.42	3.1418 (18)	142
N4—H4A···S1^ii^	0.86	2.57	3.4150 (18)	168
C18—H18B···S1^ii^	0.96	2.83	3.594 (3)	137

Symmetry codes: (i) −*x*+1, −*y*, −*z*+1; (ii) −*x*, −*y*, −*z*.
